# Evaluation of the Efficacy and Safety of Switching to Pasireotide in Patients With Acromegaly Inadequately Controlled With First-Generation Somatostatin Analogs

**DOI:** 10.3389/fendo.2019.00931

**Published:** 2020-02-03

**Authors:** Mônica Gadelha, Marie Bex, Annamaria Colao, Elier Mitsael Pedroza García, Catalina Poiana, Marisela Jimenez-Sanchez, Serkan Yener, Rishav Mukherjee, Amy Bartalotta, Ricardo Maamari, Gérald Raverot

**Affiliations:** ^1^Hospital Universitário Clementino Fraga Filho, Universidade Federal do Rio de Janeiro, Rio de Janeiro, Brazil; ^2^Department of Endocrinology, University Hospitals Leuven, Leuven, Belgium; ^3^Responsabile di Area Complessa di Endocrinologia, Università Federico II di Napoli, Naples, Italy; ^4^Hospital de la Paz, Durango, Mexico; ^5^C.I. Parhon National Institute of Endocrinology, Carol Davila University of Medicine and Pharmacy, Bucharest, Romania; ^6^Hospital de Especialidades Centro Medico Nacional La Raza IMSS, Mexico City, Mexico; ^7^Division of Endocrinology, School of Medicine, Dokuz Eylul University, Izmir, Turkey; ^8^Novartis Pharmaceuticals Corporation, East Hanover, NJ, United States; ^9^Groupement Hospitalier Est, Hospices Civils de Lyon, Lyon, France

**Keywords:** somatostatin, pasireotide, acromegaly, growth hormone, insulin-like growth factor I

## Abstract

**Introduction:** Acromegaly is a rare, serious endocrine disorder characterized by excess growth hormone (GH) secretion by a pituitary adenoma and overproduction of insulin-like growth factor I (IGF-I). Transsphenoidal surgery is the treatment of choice, although many patients require additional interventions. First-generation somatostatin analogs (SSAs) are the current standard of medical therapy; however, not all patients achieve control of GH and IGF-I. Outcomes from a Phase IIIb open-label study of patients with uncontrolled acromegaly on first-generation SSAs switching to pasireotide are reported.

**Methods:** Adults with uncontrolled acromegaly (mean GH [mGH] ≥1 μg/L from a five-point profile over 2 h, and IGF-I >1.3× upper limit of normal [ULN]) despite ≥3 months' treatment with maximal approved doses of long-acting octreotide/lanreotide received open-label long-acting pasireotide 40 mg/28 days. Pasireotide dose could be increased (maximum: 60 mg/28 days) after week 12 if biochemical control was not achieved, or decreased (minimum: 10 mg/28 days) for tolerability. Patients who completed 36 weeks' treatment could continue receiving pasireotide during an extension (weeks 36–72) when concomitant medication for acromegaly was permitted. Primary endpoint was proportion of patients with mGH <1 μg/L *and* IGF-I <ULN at week 36. Biochemical control was also assessed during the extension. Safety was assessed throughout.

**Results:** One hundred and twenty-three patients were enrolled and received pasireotide; 88 patients continued into the extension. Overall, 18 [14.6% (95% CI: 8.9–22.1)] patients achieved mGH <1 μg/L and IGF-I <ULN at week 36; biochemical control was achieved in 42.9% with screening mGH 1.0–2.5 μg/L and 6.4% with screening mGH >2.5 μg/L. For patients who entered the extension, 14.8% (95% CI: 8.1–23.9), 12.5% (95% CI: 6.4–21.3), 14.8% (95% CI: 8.1–23.9) and 11.4% (95% CI: 5.6–19.9) had mGH <1 μg/L and IGF-I <ULN at weeks 36, 48, 60, and 72, respectively. During the overall study period, most frequent investigator-reported drug-related adverse events were hyperglycemia (41.5%), diabetes mellitus (23.6%), and diarrhea (11.4%).

**Conclusions:** Switching to long-acting pasireotide provided biochemical control in some patients, which was sustained with continued treatment. Long-term safety and tolerability of long-acting pasireotide was consistent with the known safety profile. These data support long-acting pasireotide for some patients with acromegaly who are uncontrolled on first generation SSAs.

**Clinical Trial Registration:** clinicaltrials.gov, identifier: NCT02354508.

## Introduction

Acromegaly is a rare, debilitating, and chronic endocrine disorder most commonly caused by excess secretion of growth hormone (GH) from a pituitary adenoma and, subsequently, hypersecretion of insulin-like growth factor I (IGF-I) (1). Chronic exposure to elevated GH and IGF-I levels in patients with acromegaly is associated with considerable comorbidities such as metabolic dysfunction leading to an increased risk of diabetes mellitus and, if left untreated, increased mortality related to cardiovascular, cerebrovascular, and pulmonary dysfunction (2, 3). The aims of treatment are to normalize GH and IGF-I (current guidelines recommend GH <1 μg/L and age-normalized serum IGF-I levels) (4) to ameliorate the signs and symptoms of the disease, as well as to reduce mortality (5, 6). Early and successful treatment to control acromegaly could reduce exposure to GH and IGF-I, thereby improving clinical outcomes (2, 4, 7). Transsphenoidal surgery is recommended as the primary treatment of choice for most patients with acromegaly. Although surgical success is reported in many cases, ~50% of patients require additional interventions such as medical therapy in order to achieve biochemical control (3).

First-generation somatostatin analogs (SSAs; long-acting octreotide and lanreotide Autogel) are the current standard of medical care for acromegaly (4, 8). However, studies have shown that up to 70% of patients fail to achieve biochemical control with first-generation SSAs (9–17). Pasireotide is a second-generation multireceptor-targeted SSA with higher affinity for somatostatin receptor subtype 5 than octreotide and lanreotide and similar affinity for subtype 2, the two most prevalent receptors expressed on somatotroph adenomas (18). A randomized, prospective, 24-week, Phase III study (PAOLA) showed that long-acting pasireotide had superior efficacy (in achieving GH <2.5 μg/L *and* IGF-I below the upper limit of normal [ULN]) vs. continued treatment with long-acting octreotide or lanreotide in patients with uncontrolled acromegaly despite ≥6 months of treatment (19). In this 36-week, Phase IIIb study (clinicaltrials.gov ID: NCT02354508), we evaluated the efficacy—according to the strict criteria for biochemical control recommended in current treatment guidelines (GH <1 μg/L and IGF-I <ULN) (4)—and safety of long-acting pasireotide in patients with uncontrolled acromegaly despite ≥3 months of treatment with maximal approved doses of first-generation SSAs. To our knowledge, this is the first prospective study of pasireotide to assess biochemical control of acromegaly based on the more recent, strict criteria as recommended by The Endocrine Society (4).

## Methods

### Patients

Male and female patients aged ≥18 years with uncontrolled acromegaly on first-generation SSAs [evidenced by mean GH (mGH) ≥1 μg/L from a five-point profile over a 2-h period *and* IGF-I >1.3 × the age- and sex-adjusted ULN] were enrolled. Patients must have received long-acting octreotide (30 or 40 mg/28 days) or lanreotide Autogel (120 mg/28 days) for ≥3 months prior to screening. Patients were excluded if they had received concomitant treatment with other medications known to reduce GH or IGF-I levels within 3 months of screening. Patients with poor glycemic control (glycated hemoglobin [HbA_1c_] >8%), concomitant disease that could prolong the QT interval, or risk factors for torsades de pointes were excluded. The study was conducted in accordance with the Declaration of Helsinki, and the protocol was reviewed and approved by an institutional review board/independent ethics committee/research ethics board before the start of the study. All patients provided written informed consent to participate.

### Study Design

**Figure 1:**
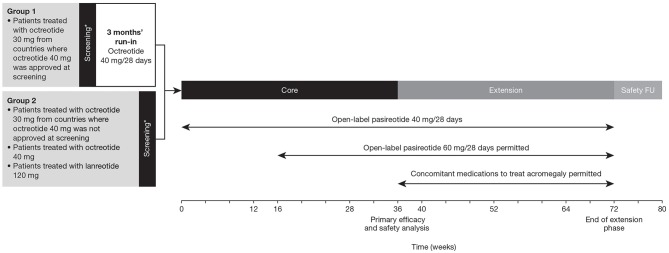
Study design. *Patients with uncontrolled acromegaly at screening (mean GH ≥1.0 μg/L and sex- and age-adjusted IGF-I >1.3 × ULN). FU, follow-up.

This was a prospective, Phase IIIb, multicenter, open-label, single-arm study comprising a 36-week core phase followed by an optional 36-week extension phase (Figure 1). Patients treated with octreotide 30 mg from countries where octreotide 40 mg was approved received the higher dose during a 3-month run-in period before being considered eligible to enter the core treatment phase. Patients who achieved biochemical control (mGH <1 μg/L *and* IGF-I <ULN) at the end of the run-in period were considered screening failures and were not eligible for the study. Patients treated with octreotide 40 mg or lanreotide 120 mg and patients treated with octreotide 30 mg from countries where octreotide 40 mg was not approved at the time of screening entered the core treatment phase directly.

Patients entering the core phase started on pasireotide 40 mg/28 days. Dose increases (maximum dose: 60 mg) were permitted at weeks 16 and 28 in patients who were uncontrolled (mGH >1 μg/L and/or IGF-I >ULN) at weeks 12 and 24, respectively, provided the lower dose was well-tolerated. The dose could be decreased (minimum dose: 10 mg) for tolerability issues and the previous dose resumed once the issue was resolved. The dose was also decreased in patients with IGF-I below the lower limit of normal *and* mGH <1.0 μg/L; the lower dose was maintained if biochemical control was achieved. Concomitant medications for acromegaly were prohibited during the core phase. Patients who completed the 36-week core phase could enter an optional 36-week extension phase and continue with the same dose of pasireotide. The dose could be increased (maximum: 60 mg) at weeks 40, 52, and 64 if patients were biochemically uncontrolled. Concomitant medications used to treat acromegaly were permitted during the extension based on the investigator's clinical judgment.

### Outcomes

The primary endpoint was the proportion of patients who achieved biochemical control (mGH <1 μg/L *and* IGF-I <ULN) at week 36. A supporting analysis assessed the primary endpoint by mGH at screening (1.0–2.5 and >2.5 μg/L). Secondary endpoints during the core and extension phases were: change in mGH and IGF-I from baseline over time, the proportion of patients with (i) mGH <1.0 μg/L and (ii) IGF-I <ULN over time, change in health-related quality of life (HRQoL) and self-reported signs and symptoms of acromegaly over time, and assessment of safety and tolerability of long-acting pasireotide.

### Assessments

Five-point mGH (collected over a 2-h period) and IGF-I levels prior to study drug administration were assessed at a central laboratory (Q2 Solutions, CA, USA) at weeks −20 (group 1 only, during screening run-in), −4 (screening), 12, 24, 36, 48, 60, and 72. GH samples were measured using a validated chemiluminescent immunometric assay (Immulite® 2000; Diagnostic Products Corp). Serum IGF-I samples were measured using a chemiluminescent assay (IDS-iSYS; ImmunoDiagnostic Systems). HRQoL was assessed using the Acromegaly Quality-of-Life Questionnaire (AcroQoL) (20) and a general health status instrument (EuroQol Five Dimensions Five Levels [EQ-5D-5L]) (21). Patients were assessed at baseline and weeks 12, 24, 36, and 72. The AcroQoL instrument comprised 22 questions divided into two scales evaluating physical aspects (8 items) and psychological aspects (14 items). Each question had a five-item Likert scale with final scores ranging from 0 (worst HRQoL) to 100 (best HRQoL). The EQ-5D-5L consisted of a descriptive system (comprising mobility, self-care, usual activities, pain/discomfort and anxiety/depression) and EuroQol visual analog scale that could range from −0.281 (worst imaginable health state) to 1 (best imaginable health state). Standardized scores for each of the acromegaly symptoms (ring size, headache, fatigue, perspiration, paresthesia, and osteoarthralgia) were reported by the patient throughout (0 = absent, 1 = mild, 2 = moderate, 3 = severe, 4 = very severe). Diabetic status of patients was assessed as follows: diabetic, patients taking antidiabetic medication or with a past medical history of diabetes mellitus, HbA_1c_ ≥6.5%, fasting plasma glucose (FPG) ≥126 mg/dL, or 2-h plasma glucose during oral glucose tolerance test (OGTT) at screening visit ≥200 mg/dL; pre-diabetic, patients with FPG 100– <126 mg/dL, HbA_1c_ 5.7– <6.5%, or 2-h plasma glucose during OGTT at screening visit 140– <200 mg/dL; non-diabetic, patients not qualifying as diabetic or pre-diabetic and with FPG <100 mg/dL, HbA_1c_ <5.7%, or 2-h plasma glucose during OGTT at screening visit <140 mg/dL. Patients monitored their own FPG by fingerstick at least three times a week for the first 4 weeks of pasireotide treatment or when the dose of pasireotide was increased; if FPG levels remained <100 mg/dL, monitoring could be decreased to two times a week between weeks 4 and 12 and once a week for the rest of the study, and then at the discretion of the investigator during the extension phase. HbA_1c_ was assessed at weeks −4 (screening), 0 (baseline), 12, 24, 36, 48, 60, and 72. Safety was assessed by monitoring adverse events (AEs). AEs were defined using the Medical Dictionary for Regulatory Activities version 20.1 and graded according to Common Terminology Criteria for Adverse Events version 4.03. The relationship of AEs to study drug was at the judgment of the investigator. Serious AEs (SAEs) were defined according to standard reporting criteria as any undesirable sign, symptom or medical condition that has one or more of the following characteristics: life threatening or fatal; necessitates/prolongs existing hospitalization; results in persistent or significant disability/incapacity; constitutes a congenital anomaly or birth defect; is medically significant (i.e., jeopardizes the patient or requires medical or surgical intervention to prevent one of the above outcomes).

### Statistical Methods

Sample size calculation was based on the primary endpoint. A sample size of 100 was selected to enable the estimation of the proportion of patients who achieved biochemical control with pasireotide at week 36 as 15%, with a precision of 7% for the associated asymptotic two-sided 95% confidence interval (CI). Considering a dropout rate of 10%, the sample size required was 112. Efficacy analyses were performed using the full analysis set (all patients who received ≥1 dose of pasireotide) for the core study and for patients who continued beyond week 36 for the extension phase. Safety analyses were performed for all patients who received ≥1 dose of pasireotide and had a post-baseline safety assessment. No formal statistical hypothesis testing was planned.

## Results

### Study Population

**Figure 2:**
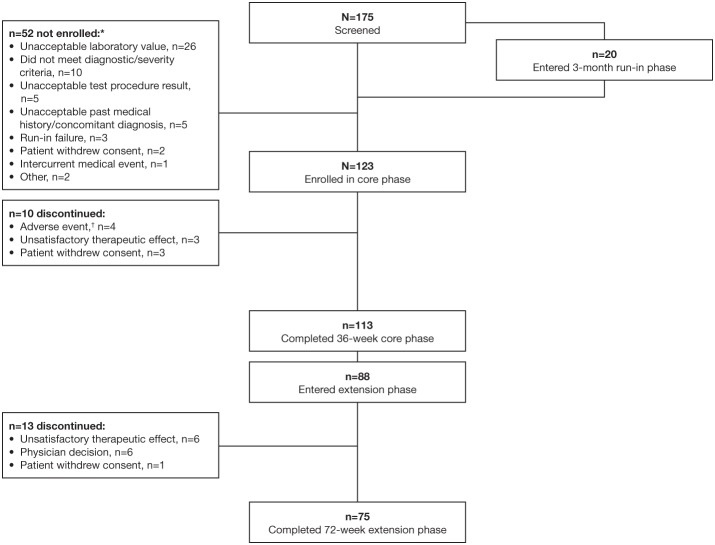
Patient disposition. *More than one reason for screening failure could be specified for each patient; ^*†*^Hyperglycemia (*n* = 3), ketoacidosis (*n* = 1), stress cardiomyopathy (*n* = 1).

Between 31 March 2015 and 12 April 2017, 175 patients were screened and 123 were enrolled and entered the core study (Figure 2). Twenty patients were receiving octreotide 30 mg in countries where the 40 mg dose was approved and therefore completed the 3-month run-in phase; of these, three patients (15%) achieved biochemical control following up-titration to octreotide 40 mg and were classified as run-in failures and not enrolled. Previous treatment received for 3–6 months prior to study start was lanreotide 120 mg (*n* = 41), octreotide 30 mg (*n* = 29), and octreotide 40 mg (*n* = 53). Most enrolled patients completed the 36-week core study (91.9%; *n* = 113); 77.9% (*n* = 88) of these patients continued into the extension phase.

At baseline, most patients (76.4%; *n* = 94) had mGH >2.5 μg/L. Most patients also had diabetes (42.3%; *n* = 52) or pre-diabetes (48.8%, *n* = 60; Table 1); 25.2% of all patients were receiving antidiabetic medication prior to the start of the study. Use of concomitant antidiabetic medications after the start of the study was reported in 62.6% of all patients, most commonly biguanides (52.0%; mainly metformin), dipeptidyl peptidase 4 inhibitors (21.1%; mainly vildagliptin and sitagliptin), and sulfonylureas (18.7%; mainly gliclazide).

**Table 1:** Baseline characteristics and demographics.

	**All patients (*N* = 123)**
Median age, years (range)	43.0 (22.0–76.0)
Female, *n* (%)	62 (50.4)
Median time since diagnosis, months (range)^*^	51.7 (1.0–405.7)
Mean mGH, μg/L (SD)^†^	10.2 (22.2)
Screening mGH stratum, *n* (%)	
1.0–2.5 μg/L	28 (22.8)
>2.5 μg/L	94 (76.4)
Missing	1 (0.8)
Mean IGF-I, x ULN (SD)^†^	2.7 (1.2)
Diabetic status, *n* (%)^‡^	
Diabetic	52 (42.3)
Pre-diabetic	60 (48.8)
Non-diabetic	11 (8.9)
Treatment prior to enrollment, *n* (%)	
Lanreotide 120 mg	41 (33.3)
Octreotide 30 mg	29 (23.6)
Octreotide 40 mg	53 (43.1)

* Three patients were enrolled and received pasireotide, who had been treated with octreotide or lanreotide for <3 months, noted as protocol deviations;

† One patient had missing mGH and IGF-I values at baseline;

‡ *Classification of patients as diabetic, pre-diabetic, or non-diabetic was performed according to multiple criteria as stated in the Methods. n, number of patients; SD, standard deviation*.

For patients who entered the extension, baseline characteristics were similar to those of the overall population (Supplementary Table 1).

### Change in mGH and IGF-I During the Core Study

**Figure 3:**
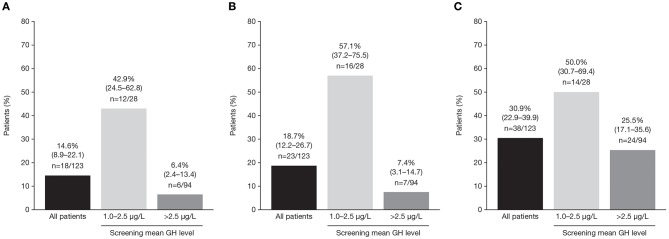
Percentage (95% CI) of patients with **(A)** mGH <1.0 μg/L *and* IGF-I <1.0 × ULN, **(B)** mGH <1.0 μg/L, and **(C)** IGF-I <1.0 × ULN at week 36, in all patients and by screening mean GH level.

**Figure 4:**
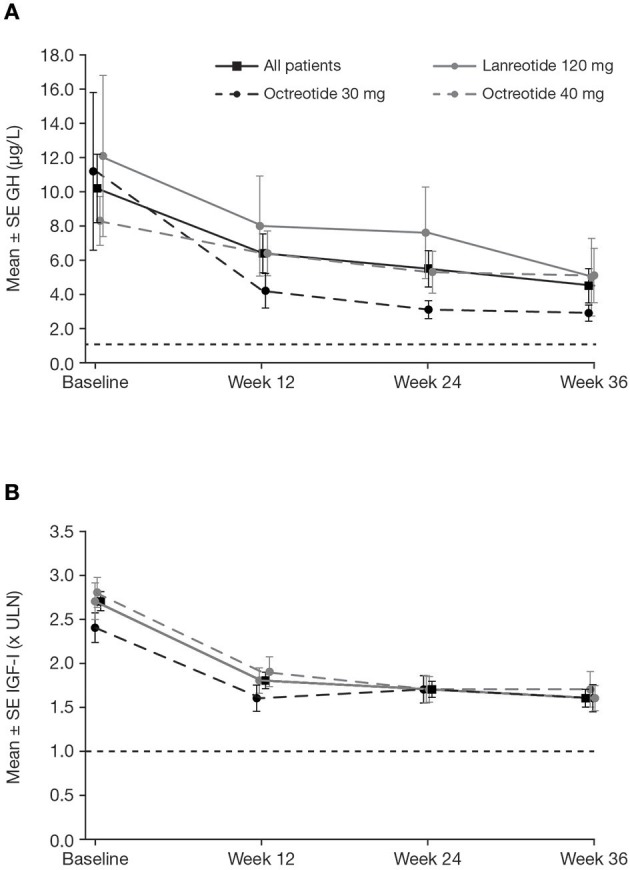
Mean ± SE **(A)** mGH and **(B)** IGF-I by visit during the core phase, in all patients and by previous first-generation SSA treatment. Reference line is **(A)** 1.0 μg/L and **(B)** ULN. SE, standard error.

In the core study, patients received pasireotide for a median of 36.0 weeks; the mean ± SD pasireotide dose was 50.0 ± 7.2 mg/month. Of patients who started treatment with 40 mg pasireotide, 90 (73.2%) were up-titrated to 60 mg after weeks 12 or 24. At week 36, 18/123 patients (14.6%; 95% CI: 8.9–22.1) achieved both mGH <1.0 μg/L *and* IGF-I <ULN (Figure 3A). Response rates were similar between patients who were previously treated with long-acting octreotide 30 mg (13.8%; 95% CI: 3.9–31.7), octreotide 40 mg (15.1%; 95% CI: 6.8–27.6), and lanreotide 120 mg (14.6%; 95% CI: 5.6–29.2). Biochemical control at week 36 was achieved by a greater proportion of patients with screening mGH 1.0–2.5 μg/L (42.9%; 95% CI: 24.5–62.8) than >2.5 μg/L (6.4%; 95% CI: 2.4–13.4%; Figure 3A). A greater proportion of patients in the lower screening mGH stratum achieved mGH <1.0 μg/L (Figure 3B) and IGF-I ≤ULN (Figure 3C), separately, at week 36. Mean mGH and IGF-I levels progressively decreased from baseline up to week 36 in all prior first-generation SSA treatment groups (Figures 4A,B).

### Change in mGH and IGF-I During the Extension Phase

**Figure 5:**
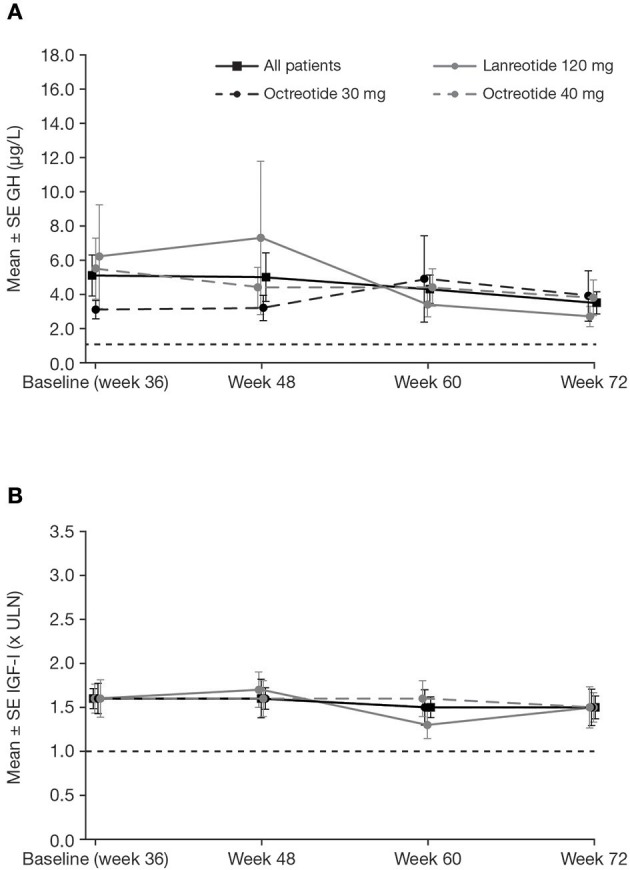
Mean ± SE **(A)** mGH and **(B)** IGF-I by visit during the extension, in all patients and by previous first-generation SSA treatment. Reference line is **(A)** 1.0 μg/L and **(B)** ULN.

Patients who completed the core and extension phases of the study received pasireotide for a median (range) of 71.9 (12–76) weeks; mean ± SD pasireotide dose was 52.5 ± 9.1 mg/month. Of the 88 patients who entered the extension phase, 70 were up-titrated to pasireotide 60 mg at any time during the study. Biochemical response rates were sustained throughout the extension (Table 2). Mean mGH and IGF-I levels were stable from week 36 to 72 in all prior first-generation SSA treatment groups (Figures 5A,B). In total, 12 patients received concomitant medication for their acromegaly (cabergoline [*n* = 10; 0.25–0.5 mg], pegvisomant [*n* = 1; 10 mg], and bromocriptine [*n* = 1; 10 mg]) during the extension phase. At week 72, none achieved both mGH <1 μg/L and IGF-I <ULN, although two patients (16.7%; 95% CI: 2.1–48.4) achieved IGF-I <ULN only.

**Table 2:** Summary of biochemical response rates in the extension study for patients who entered the extension (*N* = 88).

	**Patients**, ***n*** **(%) [95% CI]**			
	Week 36^*^	**Week 48**	**Week 60**	**Week 72**
mGH <1.0 μg/L *and* IGF-I <ULN	13 (14.8) [8.1–23.9]	11 (12.5) [6.4–21.3]	13 (14.8) [8.1–23.9]	10 (11.4) [5.6–19.9]
mGH <1.0 μg/L	19 (21.6) [13.5–31.7]	21 (23.9) [15.4–34.1]	19 (21.6) [13.5–31.7]	18 (20.5) [12.6–30.4]
IGF-I <ULN	30 (34.1) [24.3–45.0]	29 (33.0) [23.3–43.8]	33 (37.5) [27.4–48.5]	29 (33.0) [23.3–43.8]

* *End of core study/extension baseline*.

### Changes in HRQoL and Signs and Symptoms of Acromegaly

At core baseline, mean ± SD AcroQoL score was 58.6 ± 19.2 (*n* = 123), which increased to 63.2 ± 4.6 (*n* = 110) at week 36. For patients who entered the extension phase, mean ± SD AcroQoL score was 64.0 ± 19.3 (*n* = 88) at extension baseline, which increased to 65.1 ± 18.7 (*n* = 74) at week 72. Mean ± SD EQ-5D-5L index score was 0.8 ± 0.2 (*n* = 123) at core baseline and 0.9 ± 0.1 (*n* = 111) at week 36. For patients who entered the extension phase, mean ± SD EQ-5D-5L index score was 0.9 ± 0.1 (*n* = 86) at extension baseline and 0.9 ± 0.1 (*n* = 74) at week 72. Relevant changes in symptoms of acromegaly were not reported during the study. The majority of patients without symptoms at baseline continued without symptoms post-baseline. Of patients included in the core phase, the proportion of patients without symptoms at baseline vs. post-baseline, respectively, was similar: headache (41.5% vs. 36.6%), fatigue (36.6% vs. 26.0%), perspiration (43.1% vs. 37.4%), osteoarthralgia (33.3% vs. 26.8%), and paresthesia (54.5% vs. 47.2%). The proportion of patients without symptoms of acromegaly at extension baseline vs. post-extension baseline were also similar: headache (69.3% vs. 46.6%), fatigue (56.8% vs. 51.1%), perspiration (68.2% vs. 54.5%), osteoarthralgia (52.3% vs. 43.2%), and paresthesia (79.5% vs. 67.0%).

### Safety

Most patients (93.5%; *n* = 115/123) experienced at least one AE during the overall study period; 82.9% (*n* = 102/123) of patients had at least one AE that was suspected to be study drug related (Table 3). The most common investigator-assessed drug-related AEs (≥10% of patients) were hyperglycemia (41.5%), diabetes mellitus (23.6%), and diarrhea (11.4%), most of which were grade 1 or 2. No grade 4 drug-related AEs were reported, and no patient died during the study. AEs leading to study drug discontinuation were hyperglycemia (*n* = 3; 2.4%), ketoacidosis (*n* = 1; 0.8%), and stress cardiomyopathy (*n* = 1; 0.8%), all of which occurred during the core phase of the study.

**Table 3:** Most common drug-related AEs (occurring in ≥5% of patients) during the overall study period, by severity (all grades and grade 3/4).

	**All patients**, ***N*** **=** **123**	
**Adverse events**	**All grades, *n* (%)**	**Grade 3/4, *n* (%)**
Any AE	115 (93.5)	29 (23.6)
Suspected to be drug related	102 (82.9)	12 (9.8)
Any SAE	12 (9.8)	10 (8.1)
Suspected to be drug related	4 (3.3)	4 (3.3)
Any AE leading to discontinuation	4 (3.3)	2 (1.6)
Suspected to be drug related	3 (2.4)	1 (0.8)
Deaths	0	0
**Most common AEs suspected to be related to study drug (>5% based on all grades)**		
Hyperglycemia	51 (41.5)	4 (3.3)
Diabetes mellitus	29 (23.6)	5 (4.1)
Diarrhea	14 (11.4)	0
Cholelithiasis	11 (8.9)	2 (1.6)
Abdominal pain	10 (8.1)	1 (0.8)
Alopecia	9 (7.3)	0
Sinus bradycardia	8 (6.5)	0

*n, number of patients; only AEs occurring on or after the start of study treatment and no more than 3 months (84 days) after the discontinuation of study treatment of pasireotide are reported. Patients with multiple severity grades for an AE are only counted under the maximum grade*.

In total, 12 patients experienced at least one SAE; each SAE was reported in only one patient, with the exception of adrenal insufficiency (*n* = 2) and cholelithiasis (*n* = 2; Supplementary Table 2). Of these 12 patients, four experienced SAEs that were suspected to be related to pasireotide and one patient discontinued the study as a result: one patient had grade 3 abdominal pain and grade 3 adrenal insufficiency, both of which resolved with drug interruption and concomitant medication; one patient experienced grade 3 abdominal pain and cholelithiasis, both of which resolved with drug interruption and concomitant medication; one patient had grade 3 hyperglycemia and ketoacidosis, which led to study drug discontinuation; one patient experienced grade 3 cholelithiasis and biliary dilation, which remained unresolved with concomitant medication.

The most frequently reported AEs of special interest (>10% of patients overall, regardless of study drug relationship) were hyperglycemia related (*n* = 94/123; 76.4%), gallbladder/biliary related (*n* = 16/123; 13.0%), and low blood cell count related (*n* = 18/123; 14.6%). Most of the hyperglycemia- and gallbladder/biliary-related AEs were suspected by the investigator to be related to study drug; however, this was not the case for the majority of AEs related to low blood cell count.

At baseline, most patients had FPG levels 100– <126 mg/dL (*n* = 82; 66.7%), which increased to ≥126 mg/dL at the last post-baseline measurement in 43 patients (52.4%; Table 4). Overall, 7/28 patients (25.0%) with FPG <100 mg/dL at baseline had increases to ≥126 mg/dL at the last post-baseline measurement (Table 4). Most patients had HbA_1c_ levels ranging from 5.7% to <6.5% at baseline (*n* = 68, 55.3%), which increased to ≥6.5% in 33 patients at the last post-baseline measurement (48.5%; Table 4). Of patients still in the study at week 36, 46 were classified as pre-diabetic (*n* = 31 [67.4%] were pre-diabetic at baseline) and 56 as diabetic (*n* = 20 [35.7%] were pre-diabetic at baseline) according to study criteria (see Methods). At week 72, 24 patients were classified as pre-diabetic (*n* = 15 [62.5%] were pre-diabetic at baseline) and 43 as diabetic (*n* = 11 [25.6%] were pre-diabetic at baseline).

Table 4Shift in FPG and HbA_1c_ from baseline to last reported value during the overall study period.

**Table:** 

		**Last post-baseline value**, ***n*** **(%)**			
Baseline FPG, mg/dL^*^	***n* (%)**	** <100**	**100– <126**	**≥126**	**Missing**
**FPG, mg/dL**						
<100	28 (22.8)	5 (17.9)	16 (57.1)	7 (25.0)	0
100– <126	82 (66.7)	2 (2.4)	37 (45.1)	43 (52.4)	0
≥126	12 (9.8)	1 (8.3)	0	11 (91.7)	0
Missing	1 (0.8)^†^	0	0	1 (100)	0

**Table:** 

Baseline HbA_1c_, %^*^	***n*** **(%)**	** <5.7**	**5.7–<6.5**	**6.5–<8.0**	**≥8.0**	**Missing**
**HbA_1c_, %**						
<5.7	36 (29.3)	15 (41.7)	14 (38.9)	7 (19.4)	0	0
5.7–<6.5	68 (55.3)	6 (8.8)	28 (41.2)	29 (42.6)	4 (5.9)	1 (5.6)
6.5–<8.0	18 (14.6)	0	1 (5.6)	8 (44.4)	8 (44.4)	1 (5.6)
Missing	1 (0.8)^†^	0	0	0	1 (100)	0

*Includes data from the core and extension phase. Classification of patients as diabetic, pre-diabetic, or non-diabetic was performed according to multiple criteria as stated in the Methods*.

* Baseline is defined as the last available value on or before the study drug start date;

† *One patient had missing mGH and IGF-I values at baseline*.

## Discussion

Prolonged exposure to GH and IGF-I in patients with acromegaly progressively induces many systemic complications. The therapeutic goal in acromegaly is therefore to reduce morbidity and mortality by restoring GH and IGF-I levels to within the normal range as quickly as possible. This Phase IIIb, open-label study assessed the efficacy and safety of pasireotide in patients with acromegaly who had uncontrolled GH and IGF-I levels for ≥3 months despite medical treatment with first-generation SSAs. Following a revised consensus on the definition of biochemical control (mGH <1.0 μg/L and IGF-I <ULN), this is the first prospective study to assess the efficacy of pasireotide according to these strict criteria, with lower cut-off values. Even with a lower cut-off value for mGH, nearly 15% of patients who were uncontrolled for a minimum of 3 months with maximal approved doses of first-generation SSAs achieved biochemical control after the administration of pasireotide at week 36. Furthermore, biochemical response rates were sustained throughout the extension phase up to week 72. Overall, both mGH and IGF-I levels rapidly declined in the first 12 weeks and were then maintained in those patients who continued pasireotide treatment. These findings are clinically relevant as patients often require long-term or lifelong medical therapy to control their acromegaly (22). Lower GH levels at screening had a favorable impact on the achievement of biochemical control. Further investigation into biomarkers that could be used to predict responsiveness to pasireotide treatment remains of interest to optimize outcomes when considering switching patients from first-generation SSAs (23).

Not only did the current study use the more stringent recommendations for defining biochemical control than in the previous Phase III PAOLA study (19), these recommendations were also applied to the definition of patients with inadequately controlled acromegaly enrolled in the study. As such, the current study also included patients with mGH levels between 1.0 and 2.5 μg/L. Furthermore, the more rigorous IDS-iSYS assay was used for measuring IGF-I (24), compared with the Immulite assay used in the PAOLA study. Even so, our results corroborate findings from the previous Phase III study in which long-acting pasireotide provided biochemical control (according to the less strict definition of biochemical control recommended in clinical guidelines at the time: mGH <2.5 μg/L and IGF-I <ULN) (25) in 15.4–20.0% of patients who were uncontrolled for ≥6 months despite receiving first-generation SSAs (19).

Notably, during the run-in phase of our study, three patients achieved biochemical control while receiving octreotide 40 mg and therefore did not enter the core phase of the study. This highlights the role of dose optimization of first-generation SSAs in patients with uncontrolled acromegaly. In addition to suitable and timely titration of first-generation SSAs, for patients with uncontrolled acromegaly, an earlier switch to pasireotide may be considered in those who remain uncontrolled or do not tolerate maximum approved doses of first-generation SSAs.

Improvements in biochemical control would be expected to translate into improvements in HRQoL and signs and symptoms of acromegaly, as previously shown in medically naïve patients and those uncontrolled with first-generation SSAs (19, 26). Despite a trend toward improving AcroQoL scores over time, the improvement was small. Furthermore, there was no meaningful improvement in EQ-5D-5L index score or acromegaly signs and symptoms. This is likely a result of differences in the patient populations enrolled in the present study vs. those enrolled in the PAOLA study (19), a lower proportion of whom had diabetes at baseline (although a larger proportion had pre-diabetes), as well as lower mGH levels and therefore possibly less severe disease.

The AEs that were reported throughout the study were consistent with the known safety profile of long-acting pasireotide, mostly grade 1 or 2 in severity, and similar to those reported in previous Phase III studies (19, 26). The majority of AEs were related to hyperglycemia. Impaired glucose tolerance and diabetes are common comorbidities associated with uncontrolled acromegaly (27). With many patients entering the study with diabetes (42.3%) or pre-diabetes (48.8%), likely as a result of inadequate biochemical control with high-dose SSAs, hyperglycemia may have been exacerbated by pasireotide treatment (28). Hyperglycemia is manageable with proactive monitoring and early intervention with antidiabetic medications as required (28, 29). There was a low rate of patient discontinuations from the study because of AEs, with no patients discontinuing for this reason during the extension phase. This finding highlights that AEs, including those related to hyperglycemia, can be effectively managed over time.

This study has a number of limitations that we acknowledge. This was an open-label, single-arm trial in which only pasireotide was evaluated with the aim of normalizing GH and IGF-I levels in uncontrolled patients; alternative compounds were therefore not evaluated or compared with pasireotide. However, since the patients had previously been treated with first-generation SSAs with insufficient therapeutic benefit, it was not known whether pasireotide would be efficacious. The fact that a stricter criterion was used to define biochemical control highlighted the effect of pasireotide in these patients. It is also possible that continued treatment with octreotide or lanreotide might have induced biochemical control in a small proportion of patients with longer-term treatment (30); however, this exploratory study did not include a control group of patients. Furthermore, no statistical tests were conducted owing to the exploratory nature of the study. Exclusion of patients with poor glycemic control may also have induced some selection bias. A recent expert consensus statement recommends switching to pegvisomant rather than pasireotide in patients with impaired glucose metabolism and uncontrolled acromegaly on first-generation SSAs (8).

In conclusion, the results of this Phase IIIb study support the clinical benefit of long-acting pasireotide in some patients with acromegaly uncontrolled after a minimum of 3 months' treatment with maximal approved doses of first-generation SSAs.

## Data Availability Statement

Novartis is committed to sharing with qualified external researchers access to patient-level data and supporting clinical documents from eligible studies. These requests are reviewed and approved by an independent review panel on the basis of scientific merit. All data provided are anonymized to respect the privacy of patients who have participated in the trial, in line with applicable laws and regulations. This trial data availability is in accordance with the criteria and process described at www.clinicalstudydatarequest.com.

## Ethics Statement

The studies involving human participants were reviewed and approved by the ethics committees and institutional review boards listed in the Supplementary Appendix. The patients/participants provided their written informed consent to participate in this study.

## Author Contributions

The academic investigators enrolled patients in the study. RMu performed the statistical/data analyses. All authors contributed to data interpretation and writing, reviewing, and amending of the manuscript, made the decision to submit the manuscript for publication, and vouch for the accuracy and completeness of the data.

### Conflict of Interest

The authors declare that this study was funded by Novartis Pharma AG. The study was designed by the academic investigators and the sponsor, Novartis Pharma AG. Data were collected by investigators using Novartis' data management systems and analyzed by Novartis' statistical team. The first draft was prepared by a medical writer funded by Novartis Pharmaceuticals Corporation. MG: Advisory board member: Novartis Pharmaceuticals. Research investigator: Novartis Pharmaceuticals, Ipsen. Speaker: Novartis Pharmaceuticals, Ipsen, Pfizer Inc. MB: Research investigator: Novartis Pharmaceuticals. AC: Grant recipient: Novartis Pharmaceuticals. MJ-S: Research investigator: Sanofi Pharmaceuticals. RMu: Employee: Novartis Healthcare Pvt Ltd. AB: Employee: Novartis Pharmaceuticals. RMa: Employee: Novartis Pharmaceuticals. GR: Advisory board member: Novartis Pharmaceuticals, Pfizer Inc. Research investigator: Novartis Pharmaceuticals, Ipsen. Speaker: Novartis Pharmaceuticals, Ipsen, Pfizer Inc. The remaining authors declare that the research was conducted in the absence of any commercial or financial relationships that could be construed as a potential conflict of interest.
