# Supplementary material for: Evaluation of the Efficacy and Safety of Switching to Pasireotide in Patients With Acromegaly Inadequately Controlled With First-Generation Somatostatin Analogs
Source: Front Endocrinol (Lausanne). 2020 Feb 3;10:931. doi: 10.3389/fendo.2019.00931 (PMC7008501; doi:10.3389/fendo.2019.00931)
Supplement: Supplementary file 1 [file Data_Sheet_1.pdf]

Evaluation of the Efficacy and Safety of Switching to Pasireotide in Patients with Acromegaly Inadequately Controlled with First-Generation Somatostatin Analogues

Supplementary Appendix

List of independent ethics committees or institutional review boards from participating centers in the study that provided ethics approval

Consejo Institucional de Revision de Estudios De Investigacion (Buenos Aires, Argentina), Comite d'Ethique hospital-facultaire, UCL Saint-Luc (Brussels, Belgium), Ethische commissie onderzoek UZ Leuven (Leuven, Belgium), Ethisch Comite UZA (Antwerpen, Belgium), Comite de Etica em Pesquisa do Hospital Universitario Clementino, Fraga Filho – HUCFF – UFRJ (Rio de Janeiro, Brazil), Ethics Committee for Multicenter Trials, Bulgarian Drug Agency (Sofia, Bulgaria), Peking Union Medical College Hospital of the Chinese Academy of Medical Sciences – Ethics Committee on Drug Clinical Trials (Beijing, China), The First Affiliated Hospital, Sun Yat-sen University – Ethics Committee on Clinical Medicine, Equipment and New Medical Technology (Guangdong Province, China), Comite de etica en Investigacion CEI-FOSCAL (Santander, Colombia), CPP Sud-Est IV (Lyon, France), ETT Egeszsegugyi Tudomanyos Tanacs (Budapest, Hungary), Comitato Etico Milano area 2 (Milano, Italy), Comitato Etico Universita' Federico II di Napoli (Napoli, Italy), Comitato Etico per la Sperimentazione Clinica Della Provincia di Padova (Padova, Italy), Comitato Etico Palermo 1 (Palermo, Italy), Comitato Etico Interaziendale AO Citta' Della Salute e Della Scienza di Torino (Torino, Italy), Comitato Etico Regionale Della Liguria (Genova, Italy), Comitato Etico Milano area 3 (Milano, Italy), Comitato Etico Regionale (CER) Delle Marche (Ancona, Italy), Comitato Regione Toscana – Area Vasta Nord Ovest (Pisa, Italy), Medical Research and Ethics Committee (Kuala Lumpur, Malaysia), Comite de Etica en Investigacion del Instituto Mexicano del Seguro Social, Coordinacion de Investigacion en Salud (Mexico City, Mexico), Comite de Etica en Investigacion del Instituto Jalisciense de Investigacion Clinica, SA de CV (Guadalajara, Mexico), Comite de Etica en Investigacion de la Clinica Bajiso CLINBA, SC (Guanajuato, Mexico), Comissão de Ética para a Investigação Clínica (Lisboa, Portugal), Comisia Nationala de Bioetica a Medicamentului si a Dispozitivelor Medicale (Bucuresti, Romania), Kocaeli University Clinical Research Ethics Committee (Kocaeli, Turkey), NRES Committee London-Fulham (Manchester, UK)

Supplementary Table 1. Extension patient population, baseline characteristics and demographics

	All patients (N=88)
Median age, years (range)	43.0 (22.0–76.0)
Female, n (%)	47 (53.4)
Median time since diagnosis, months (range)*	51.7 (1.0–405.7)
Mean mGH, µg/L (SD)	11.4 (25.8)
Screening mGH stratum, n (%)	
1.0–2.5 µg/L	20 (22.7)
>2.5 µg/L	68 (77.3)
Missing	0
Mean IGF-I, x ULN (SD)	2.7 (1.2)
Diabetic status, n (%)†	
Diabetic	43 (48.9)
Pre-diabetic	36 (40.9)
Non-diabetic	9 (10.2)
Treatment prior to enrollment, n (%)	
Lanreotide 120 mg	26 (29.5)
Octreotide 30 mg	23 (26.1)
Octreotide 40 mg	39 (44.3)

*Three patients were enrolled who had been treated with octreotide or lanreotide for <3 months, noted as protocol deviations; †Classification of patients as diabetic, pre-diabetic or non-diabetic was performed according to multiple criteria as stated in the Methods. FPG, fasting plasma glucose; HbA_{1c}, glycated hemoglobin; IGF-I, insulin-like growth factor I; mGH, mean growth hormone; OGTT, oral glucose tolerance test; SD, standard deviation; ULN, upper limit of normal

Supplementary Table 2. Summary of all serious adverse events regardless of study drug relationship during the overall study period, by severity (all grades and grade 3/4)

	**All patients, N=123**	
**Preferred term**	**All grades, n (%)**	**Grade 3/4, n (%)**
**Total**	**12 (9.8)**	**10 (8.1)**
Adrenal insufficiency	2 (1.6)	2 (1.6)
Cholelithiasis	2 (1.6)	2 (1.6)
Abdominal pain	1 (0.8)	1 (0.8)
Bile duct stone	1 (0.8)	0
Biliary dilatation	1 (0.8)	1 (0.8)
Breast cancer	1 (0.8)	1 (0.8)
Dermatofibrosarcoma protuberans	1 (0.8)	0
Diarrhea	1 (0.8)	1 (0.8)
Hyperglycemia	1 (0.8)	1 (0.8)
Ketoacidosis	1 (0.8)	1 (0.8)
Neutropenia	1 (0.8)	1 (0.8)
Nose deformity	1 (0.8)	0
Stress cardiomyopathy	1 (0.8)	1 (0.8)
Tonsillitis	1 (0.8)	1 (0.8)

n represents number of patients. A patient with multiple occurrences of an AE under one treatment is counted only once in the AE category for that treatment. A patient with multiple AEs is counted only once in the 'Total' row. AE, adverse event
